# The Functional Roles of IL-33/ST2 Axis in Ocular Diseases

**DOI:** 10.1155/2020/5230716

**Published:** 2020-08-18

**Authors:** Yujing Qian, Meifen Zhang

**Affiliations:** Department of Ophthalmology, Peking Union Medical College Hospital, Chinese Academy of Medical Sciences and Peking Union Medical College, 100005 Beijing, China

## Abstract

Interleukin-33 (IL-33), an important member of the IL-1 family, plays a pivotal role in regulating immune responses via combining with its receptor suppression of tumorigenicity 2 (ST2). We have already known IL-33/ST2 axis participates in the pathogenesis of various diseases, including liver diseases, renal diseases, and neurological diseases. Recently, emerging studies are indicating that IL-33/ST2 is also involved in a wide range of ocular diseases, such as allergic eye disease, keratitis and corneal regeneration, dry eye disease, uveitis, vitreoretinal diseases, and neuromyelitis optica spectrum disorder. In this review, we will summarize and discuss the current understanding about the functional roles of IL-33/ST2 in eyes, with an attempt to explore the possible study perspectives and therapeutic alternatives in the future.

## 1. Introduction of the IL-33/ST2 Axis

### 1.1. Expression of the IL-33 and ST2

Interleukin-1 (IL-1) family, consisting of 11 cytokines, plays a critical role in immune regulation and inflammation mediation. Interleukin-33 (IL-33, previously named as NF-HEV, IL-1F11) [1, 2] was firstly introduced as a new member of the IL-1 family in 2005 [3]. It is widely expressed in the nucleus of endothelial cells in most human tissues [4, 5]. It is also expressed in other cell types, including fibroblasts, epithelial cells, smooth muscle cells, macrophages, and dendritic cells in several organ tissues [3, 6–9]. Different from traditional cytokines, IL-33 is considered as a dual function protein, which exerts its pro- or anti-inflammatory effect by acting as both an intracellular nuclear factor and a cytokine [10]. IL-33 inside the nuclear can dock at the nucleosomal surface and promote nucleosome-nucleosome interactions and regulate chromatin compaction [11, 12]. Additionally, nuclear IL-33 can also sequester the transcription factor nuclear factor kappa B (NF-*κ*B), which results in reduced NF-*κ*B-triggered gene expression and therefore dampen the proinflammatory signaling [13]. Moreover, cytokine IL-33 can be released outside the cells and mediate immune responses via binding to its receptor.

As the specific receptor of IL-33, ST2 (suppression of tumorigenicity, also known as Fit-1, T1) is one of the members in IL-1 receptor family, coded by gene interleukin 1 receptor like 1. It was originally presented in 1989 as a serum-inducible secreted protein in mouse fibroblast [14–16]; then, the signaling complex of ST2 and IL-1R accessory protein (IL-1RAcP) was discovered. ST2 exists on a wide spectrum of cell types, such as mast cells, endothelial cells, innate lymphoid cells group 2 (ILC2s), helper and regulatory T cells, CD8+ T cells, invariant natural killer T (iNKT) cells, natural killer (NK) cells, basophils, and eosinophils [17–20]. So far, studies have discovered three variants of ST2 protein due to alternative splicing in human, namely, a transmembrane form (ST2L), a soluble secreted form (sST2), and a novel isoform (ST2V) [21, 22]. Among which, ST2L was selectively expressed on the surface of T helper 2 (Th2) cells and mast cells, but not on that of Th1 or regulatory T (Treg) cells, while sST2 was secreted by Th2 cells following signaling activation [23, 24]. The expression and roles of ST2V remain unclear.

### 1.2. Molecular Pathways of IL-33/ST2 Axis

IL-1 family members are mostly synthesized as precursor proteins with little or no biological activity, requiring limited protease to process cleavage and unlock their full biological potential [25]. However, full-length pro-IL-33 exhibits basal activity in its immature unprocessed form, which is similar to IL-1*α*, but different from IL-1*β* and IL-18. Moreover, some inflammatory proteases, such as neutrophil elastase, cathepsin G, and mast cell chymase, could greatly enhance IL-33 bioactivity via cleaving at the N-terminal part [26, 27]. In contrast, some proteases including caspase-1, and proapoptotic caspases-3 and -7 could inactivate IL-33 by cutting at its C-terminal IL-1-like cytokine domain [28, 29].

When cells are damaged or necrotic, bioactive pro-IL-33 is released from cells, and the abovementioned proteases remove the N-terminal amino acids; then, a mature, 10- to 30-fold-increased-potent IL-33 is created. Once the active IL-33 is released, it can bind to cells that express ST2L [30] and recruit IL-1RAcP. The Toll-interleukin 1 receptor (TIR) domain in IL-1RAcP then combines with a signaling adaptor including myeloid differentiation primary response protein 88 (MyD88), IL-1R-associated kinase 1 (IRAK1), IRAK4, and tumor necrosis factor receptor-associated factor 6 (TRAF6) [31]. Subsequently, the axis activates the transcription factor NF-*κ*B and the mitogen-activated protein kinases (MAPK) [21], which in turn displays profound immunomodulatory functions.

The IL-33/ST2L axis also has a negative feedback system as soluble ST2 and soluble IL-1RAcP complex can reversely regulate and even prevent the IL-33 activity [33]. Moreover, extracellular environment [34], single immunoglobulin domain IL-1R-related molecule [35], and inflammatory proteases [36] can also diminish IL-33 activity.

### 1.3. Functional Roles of the IL-33/ST2 Axis

IL-33/ST2 axis could affect cells both of the innate and adaptive immune system [37].

First, IL-33/ST2 plays critical roles in the differentiation and functionality of various T cell subsets and primarily mediates type 2 immune action. It is well recognized that IL-33 drives the polarization of naïve T cells to Th2 immune cells and induces the production of Th2 cytokines and chemokines, such as IL-5, IL-9, and IL-13 [38]. In addition, IL-33/ST2 could promote the expansion and function of both CD8+ cytotoxic T lymphocytes and Th1 cells and therefore mediate a protective antiviral response [39, 40]. The axis also provokes a Th17 immune response [41]. Besides, IL-33/ST2 displays dual functions in Treg cells in different disease contexts. Under autoimmune, inflammatory and tissue injured circumstances, such as experimental autoimmune encephalomyelitis, graft versus host disease (GVHD), sepsis, and acute lung injury, IL-33/ST2 axis increases Treg cells numbers and enhances its protective function [42–44]. However, in allergic airway disease and systemic sclerosis, IL-33/ST2 can dysregulate lung and skin Treg cells and impair its suppressive ability, respectively [45, 46].

Second, IL-33/ST2 acts on a wide range of innate immune cells [42, 47]. Specifically, IL-33/ST2 promotes the infiltration and activation of neutrophils, eosinophils, basophils, dendritic cells, iNKT, and NK cells, resulting in the release of cytokines and chemokines accordingly. IL-33/ST2 also stimulates the maturation of mast cells [48], and facilitates the polarization of M2 macrophages [49]. Furthermore, IL-33/ST2 also influences the activation of innate immune responses via the induction of ILC2s [50–53]. Consequently, IL-33/ST2 axis is categorized as an “alarmin” due to its ability to initiate both adaptive and innate immune defences in the context of cell damage [4, 20, 54].

### 1.4. Relationships between the IL-33/ST2 Axis and Diseases

Due to the comprehensive and pleiotropic involvement of the IL-33/ST2 axis in immune responses, it has been implicated in a broad range of diseases and suggested as a potential biomarker for predicting disease severity and activity [47]. Herein, we divided these diseases into two groups, according to whether they are exacerbated or ameliorated by IL-33/ST2 signaling.

In the basis of current understanding, the IL-33/ST2 pathway primarily plays detrimental roles in various allergic disorders [55, 56] such as asthma and atopic dermatitis and in multiple autoimmune diseases [47, 57] such as rheumatoid arthritis, systemic lupus erythematosus, systemic sclerosis, and inflammatory bowel diseases [58]. IL-33/ST2 also aggravates chronic obstructive pulmonary disease [59] and fibrotic diseases [60, 61].

On the contrary, IL-33/ST2 exerts protective effects on GVHD [62, 63], cardiovascular diseases [64], and several central nervous system diseases [10, 65] such as Alzheimer's disease and stroke. In addition, the axis also promotes tissue repairment [66].

However, there are still controversies since the IL-33/ST2 axis presents dichotomous roles in a number of diseases. For instance, the exacerbated or ameliorative effect of the axis during infection (e.g., bacterial, fungal, parasitic, and viral infection) depends on factors such as the infectious agents, involved organs, and cytokine microenvironments [67, 68]. Similarly, IL-33/ST2 shows protumoral or antitumoral functions up to the tumor types, target cells, and microenvironmental factors [69, 70].

## 2. Functional Roles of IL-33/ST2 in Ocular Diseases

**Figure 1:**
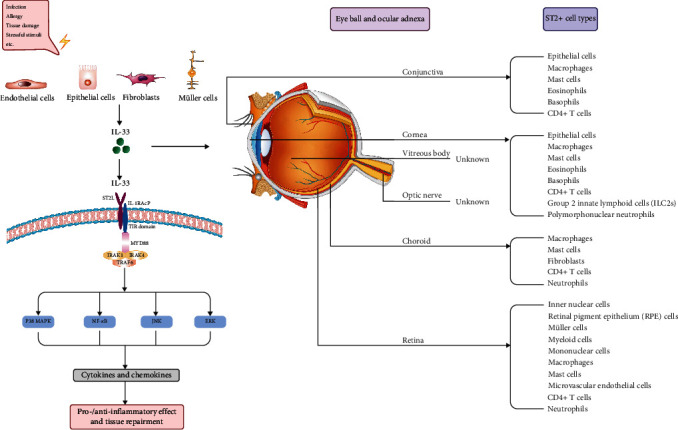
Signaling and roles of IL-33/ST2 in eye diseases. Various factors (infection, allergy, tissue damage, and stressful stimuli, etc.) result in the release of interleukin-33 (IL-33) from the nucleus of endothelial cells, epithelial cells, fibroblasts, and Müller cells. Binding of IL-33 with heterodimer receptor suppression of tumorigenicity 2 (ST2L)/IL-1R accessory protein (IL-1RAcP) induces myeloid differentiation primary response protein 88 (MyD88) recruitment via Toll-interleukin 1 receptor (TIR) domain and downstream activation involving IL-1R-associated kinase 1 (IRAK1), IRAK4, and tumor necrosis factor receptor-associated factor 6 (TRAF6). Subsequently, the p38-activated mitogen-activated protein kinases (MAPK), c-Jun N-terminal kinase (JNK), extracellular regulated protein kinases (ERK), and nuclear factor kappa B (NF-*κ*B) promote the secretion of inflammatory cytokines and chemokines, which in turn exerts pro-/anti-inflammatory effect and tissue repairment. Upregulated IL-33 in different parts of eyeball and ocular adnexa induces diverse immune responses and diseases by targeting various ST2+ cells.

Recently, the relationship between IL-33/ST2 axis and eye diseases has aroused increasing attention. Accumulating studies have demonstrated that IL33/ST2 plays critical roles in several eye diseases (Figure 1), including allergic eye disease, keratitis and corneal regeneration, dry eye disease (DED), uveitis, vitreoretinal diseases, and neuromyelitis optica spectrum disorder (NMOSD). Herein, we summarized the study progress in the abovementioned areas (Table 1).

### 2.1. Allergic Eye Disease

Allergic eye disease is a wide spectrum of ocular disorders that encompasses keratoconjunctivitis, allergic conjunctivitis, blepharoconjunctvitis, and giant papillary conjunctivitis, etc. Amongst atopic keratoconjunctivitis (AKC) is a typical Th2-biased chronic inflammatory disease, involving both conjunctival inflammation and cornea impairment, which may result in severe vision loss, and is associated with atopic dermatitis and asthma in most cases [71]. Meanwhile, allergic conjunctivitis (AC) is one of the most common hypersensitivity eye diseases characterized by itching, redness, lid edema, tearing and other signs [72]. Patients with AC typically undergo two stages after exposure to allergens, which are an IgE-dependent prompt response and a Th2 cytokine-dependent delayed response [73]. Since the 1990s, it has been widely recognized that IL-1 plays regulatory roles in AC. Considering the homologous sequences and similar receptor sharing between IL-33 and IL-1, accumulating studies have paid attention to the function of IL-33/ST2 in allergic eye diseases, especially in AKC and AC.

In patients with atopic keratoconjunctivitis, IL-33 protein expressed at vascular endothelial cells, epithelium cells, and fibroblasts of giant papillae in vivo [74]. Studies further analyzed the induction and downstream signals of IL-33/ST2 in vitro. In human corneal epithelial cells (HCECs), it was revealed that the signaling pathway was initiated by the combination of various viral or bacterial components with Toll-like receptors (TLR3, -4, -5, -6, and -7), while was largely blocked by MyD88 inhibitory peptide or TIR domain-containing adaptor-inducing interferon *β* inhibitory peptide [75]. The activated IL-33/ST2 axis can in turn induce proallergic inflammation and promote the expression of proallergic cytokine thymic stromal lymphopoietin and chemokine (C-C motif) ligand-2 (CCL2), CCL20, and CCL22 on both mRNA and protein level in the human cornea. Additionally, the signaling pathway can be significantly stimulated by IL-33, while suppressed by ST2 antibodies, soluble ST2, NF-*κ*B activation inhibitor, and inhibitor of NF-*κ*B-*α* (I*κ*B-*α*) inhibitor [76]. In mast cells, it was proved that the IL-33/ST2 exerted its functional roles via the phosphorylation of p38 MAPK and IL-13 mRNA induction [74]. To verify the in vivo pathogenic roles of IL-33/ST2 in AKC, one study group established a transgenic mouse line, in which IL-33 is overexpressed in keratinocytes [77]. Results displayed that transgenic mice spontaneously developed blepharitis and keratoconjunctivitis strikingly resembling human AKC. The upregulated level of IL-33 enhanced Th2 cytokine (IL-4, IL-5, and IL-13) expression and induced eosinophils, mast cells, and basophils infiltration in mice conjunctiva and cornea. Furthermore, in the lacrimal fluid, the concentration of IL-33, Th2 cytokines, and chemokines (CCL2, CCL3, CCL5, CCL11, C-X-C motif-binding chemokine 1, and granulocyte-colony stimulating factor) were also abundant. Interestingly, this study also demonstrated that IL-33/ST2 promoted the activation and proliferation of ILC2, resulting in markedly elevated levels of ILC2-producing IL-5, IL-13, and chemokines in the cornea. Therefore, it is obvious that the IL-33/ST2 axis participates in the initiation and development of atopic keratoconjunctivitis in several conjunctival and corneal cell types.

As for allergic conjunctivitis, current studies were conducted mainly based on various mice models. In AC mice, a significant increase of IL-33 mRNA and protein was observed in the conjunctival epithelial cells [78, 79]. The IL-33 stimulation augmented the expression of ST2 and IL-1RAcP in the conjunctiva and promoted Th2 cells differentiation [80]. Consequently, increased release of Th2 cytokines in conjunctiva and corneal epithelium triggered the infiltration of a large amount of ST2+ eosinophils and a small proportion of ST2+ CD4+ T cells [73] and ST2+ mast cells in conjunctiva, leading to an increase in total IgE concentration in serum [81]. On the contrary, in TLR4 deficient, MyD88 knockout [80] or IL-33 knockout mice [78, 79], clinical manifestation, eosinophils and basophils infiltration, and molecular concentration (protein and mRNA levels of IL-33, ST2, IL1RAcP, and Th2 cytokines) were markedly diminished or eliminated in the conjunctiva compared to those in wild-type mice. Therefore, we may conclude that IL-33/ST2 plays a promotive role in allergic conjunctivitis. Based on these present findings, further studies are necessary to investigate the possibility of IL-33/ST2-target therapeutic strategy in allergic eye disease.

### 2.2. Keratitis and Corneal Regeneration

IL-33/ST2 axis also participates in balancing the corneal inflammation with organism elimination and cell apoptosis with tissue repair.

Keratitis is a series of corneal inflammation diseases that is caused by a variety of pathogens. *Pseudomonas aeruginosa* (*P. aeruginosa*) keratitis is one of the most common and devastating bacterial keratitis, which is closely associated with extended contact lens usage [82]. In this regard, the primary explorations of the corneal localization and functional roles of ST2 [83] and IL-33 [84] were separately reported using *P. aeruginosa* keratitis mice models. Huang et al. found ST2 and IL-33 mRNA and protein constitutively expressed in the normal cornea, while the expression level of ST2 and IL-33 was significantly increased in the *P. aeruginosa*-infected cornea. Intriguingly, when treated C57BL/6 mice (susceptible) with recombinant murine (rm) IL-33 before infection, upregulated ST2 expression was observed, which then led to a reduction of bacterial load and polymorphonuclear neutrophil infiltration and decreased mRNA levels for proinflammatory cytokines (IL-1*β*, human macrophage inflammatory protein 2, IL-6, and tumor necrosis factor-*α*) and Th-1 cytokines (interferon-*γ* and IL-12), together with upregulated mRNA levels for Th-2 cytokines (IL-4, IL-5, and IL-10) in the cornea. Moreover, rmIL-33 injection also shifted macrophage polarization from M1 to M2 phenotype. Therefore, it is suggested that IL-33 stimulation resulted in less disease severity and reduced cornea inflammation [84]. However, when tested BALB/c (resistant) mice with rmST2 as a decoy receptor before infection, decreased level of activated ST2 led to converse molecular changes, and ultimately showed severer disease manifestations [83]. Taken together, these two studies innovatively revealed the potential defensive role of both IL-33 and ST2 in bacterial keratitis.

In the case of fungal infection, *Aspergillus fumigatus* could greatly provoke IL-33 expression in human corneal tissues, mice corneas, and HCECs. Subsequently, the activation of IL-33/ST2/p38 MAPK axis promoted the production of proinflammatory cytokines [85]. Therefore, current evidence indicated that the IL-33/ST2 axis may exert a detrimental effect on corneal fungal infection.

Moreover, in cultured primary HCECs, it was revealed that additional IL-33 can stimulate IL-33/ST2 signaling and increase the concentration of inflammatory mediators. While the IL-33/ST2 axis was suppressed by ST2 antibody, soluble ST2, p38 MAPK inhibitor, NF-*κ*B activation inhibitor, and I*κ*B-*α* inhibitor [85, 86]. Collectively, it is of great importance to further investigate the double-edge functions and regulatory mechanisms of IL-33/ST2 in different types of keratitis and explore clinical strategies based on diverse infectious pathogens and immune responses.

Apart from corneal infection, corneal injury is another major cause of blindness worldwide. Understanding the mechanism of cell proliferation and tissue healing is critical to restoring vision. At a steady state, only a rare population of ILC2s was detected to reside in mice corneal limbus. However, once corneal epithelium was abraded, CD64+CCR2- M2 type macrophages started to express IL-33 in the cornea, and thereby, local induction of IL-33 promoted significant ILC2s expansion and responses, resulting in corneal wound healing. In contrast, blocking of IL-33 or depleting of CCR2- macrophages can greatly delay and attenuate the process [87]. In addition, IL-33/ST2 was proved to promote cell proliferation in HCECs in vitro [85]. In summary, limited study has shed light on the beneficial roles of IL-33/ST2 in wound healing and corneal regeneration. More studies are required to elucidate the detailed regulatory pathways involving more cell types and focus on its potential clinical use in treating various corneal defective diseases.

### 2.3. Dry Eye Disease

In recent years, dry eye disease has aroused great attention due to its high prevalence and significant impact on visual quality. It is mainly categorized into two types, namely, aqueous deficient type and evaporative continuum type [88]. Nevertheless, ocular surface inflammatory response is a key component in the pathogenesis of all subtypes of DED.

In human conjunctival epithelial cells, the level of IL-33 mRNA and protein was found to be significantly enhanced in the hyperosmotic state [89]. In the conjunctival impression cytology specimens obtained from DED patients, both IL-33 and ST2 protein were increased, compared to those in healthy people. Furthermore, in tears of DED patients, the concentration of IL-33 was greatly elevated, together with various proinflammatory cytokines and chemokines [90]. The elevated IL-33 level was positively correlated with increased Th2 cytokines (L-4, IL-5, and IL-13), ocular surface disease index score, and corneal fluorescein staining, whereas negatively associated with tear film breakup time and Schirmer I test [89, 91]. Interestingly, although the abovementioned results were observed both in Sjögren syndrome dry eye and non-Sjögren syndrome dry eye patients, tear IL-33 level was found to be much higher in Sjögren syndrome group than in the non-Sjögren syndrome group [91].

Thus far, present information has interpreted that the upregulated IL-33/ST2 might mediate the ocular surface inflammation in DED via the activation of Th2 responses. The relationship between IL-33 expression levels and disease severity may also provide us a novel target for DED diagnosis and treatment. It is valuable to perform continued research including identifying the regulatory pathways of IL-33/ST2 in DED, distinguishing the differences of IL-33/ST2 expression between the aqueous deficient and the evaporative dry eye and demonstrating the correlation of IL-33/ST2 with DED-related ocular surface diseases such as corneal superficial punctate keratopathy.

### 2.4. Uveitis

Uveitis is a wide spectrum of inflammatory disease involving anterior or/and posterior of the eyeball and can result in permanent visual impairment. Although it is well-recognized that IL-1 family plays crucial roles in mediating innate and adaptive immune response and could be a potent therapeutic target in various autoimmune diseases [92], little was known about the role of IL-1 family member IL-33 and its receptor ST2 in uveitis, especially in autoimmune uveitis. IL-33 was constitutively expressed in the inner nuclear cells of normal retina and was greatly upregulated in autoimmune uveitis mice. In addition, in ST2 knockout mice, the exacerbated immune response can be reversely regulated by IL-33 treatment, which presented as promoted macrophages polarization, altered cytokine production profiles, and ultimately reduced disease severity [93]. There were also several clinical researches regarding different subtypes of uveitis. In patients with Behçet's uveitis, serum IL-33 level was significantly higher compared to patients without uveitis [94]. In a Chinese population with acute anterior uveitis (AAU), a single-nucleotide polymorphism-rs3773978 in gene interleukin 1 receptor like 1 (encodes ST2) was found to associate with the presence of disease [95]. However, different results were obtained in another study conducted in AAU patients: even though multiple cytokines of IL-1 family exhibited as the specific signatures in HLA-B27 associated AAU and idiopathic AAU, no significant differences of IL-33 level in both serum and aqueous humor were observed between disease group and control group [96]. Hence, whether the IL-33/ST2 axis involves in regulating uveal inflammation reaction and by what regulatory networks are still uncertain. Future investigations could be taken to reevaluate the role of IL-33/ST2 among more kinds of uveitis, compare the difference between infectious versus noninfectious uveitis, or granulomatous versus nongranulomatous uveitis, and clarify potential reasons for the discrepancy.

### 2.5. Vitreoretinal Diseases

The current studies concerning the roles of IL-33/ST2 axis in vitreoretinal diseases mainly focused on ocular toxoplasmosis (OT) and age-related macular degeneration (AMD). Notably, its roles in polypoidal choroidal vasculopathy (PCV), retinal detachment (RD), retinopathy of prematurity (ROP), and proliferative diabetic retinopathy (PDR) have been uncovered lately.

Ocular toxoplasmosis is a vision-threatening disease caused by *Toxoplasma gondii* infection, which generally involves vitreous body, retina, and choroid. In the eyes of *Toxoplasma gondii*-infected mice, IL-33-positive cells increased, accompanied with enhanced mRNA concentration of IL-33, ST2, IL-1*β*, Th1 (IFN-*γ* and IL-12), and Th2 (IL-4, IL-10, and IL-13) cytokines. The increased IL-33/ST2 signaling was closely related to the higher expression levels of these inflammatory mediators [97]. The same research group further revealed that in the damaged retina and choroid of OT mice, the mRNA levels of triggering receptor expressed on myeloid cells-1 and TLRs also significantly elevated and were positively correlated with the enhanced IL-33/ST2 signaling [98]. Therefore, current data indicates that IL-33/ST2 participates in the immune responses of OT and may exert its regulatory roles by indeterminate signaling pathways involving triggering receptor expressed on myeloid cells-1, TLRs, and Th1/Th2 cytokines.

Age-related macular degeneration is another disease where IL-33/ST2 might involve in [99]. AMD is one of the preponderant causes of severe visual loss in the ageing population worldwide. The main characteristics of AMD include permanent loss of photoreceptors and retinal pigment epithelium (RPE) cells, formation of drusen, and choroidal neovascularization [100].

Primarily, IL-33 was inferred as a pathogenic factor in aggravating retina injury and noninfectious immune responses. In normal human retina tissue, IL-33 was highly expressed in the nucleus of Müller cells and RPE cells of macular area. IL-33 was also positive in astrocytes in the retinal ganglion cell layer and choroidal endothelial cells [101]. In AMD patients, IL-33+ Müller cells increased in the RPE and photoreceptor loss area, which is analogous to the lesion in geographic atrophy or advanced dry AMD. IL-33+ cells also enhanced in the choroid of AMD lesion areas. Furthermore, a higher IL-33 concentration in vitreous was observed. Next, the functional roles of IL-33/ST2 signaling in RPE cells or Müller cells were identified using an AMD cell line in vitro [102] or a phototoxic retinal mice model in vivo [101], respectively. Specifically, IL-33 was released by RPE cells and Müller cells under stressful stimuli, leading to an increase in ST2L expression and recruitment of myeloid cells. The binding of bioactive IL-33 and ST2L thereby activated downstream pathways (p38 MAPK, c-Jun N-terminal kinase, extracellular regulated protein kinases 1/2, and NF-*κ*B), and induced the secretion of proinflammatory cytokines (IL-6, IL-8, IL-1*β*, and tumor necrosis factor-*α*, respectively) and chemokines. On the other hand, IL-33/ST2 also directly recruited massive mononuclear phagocytes to the outer layers of the retina. Thus, these two pathogenic functions of IL-33/ST2 may ultimately lead to inflammatory injuries and photoreceptor cell loss in AMD. Besides, after treating neovascular AMD mice with antiendoglin and/or antivascular endothelial growth factor-A antibody, a significant reduction of IL-33 production, along with attenuated development of retinal vascular lesions and subretinal fibrosis, was observed [103].

However, although various factors such as autophagy and phototoxic and oxidative stress might aggravate retina injury and noninfectious immune responses, there were still arguments addressed that IL-33 attenuated this responses [104]. According to a study by Theodoropoulou et al., hypoxic conditions or activation of Toll-like receptor might enhance glycolytic metabolic in RPE cells, which resulted in upregulated activity level of IL-33/ST2 and subsequently reduced the migration of choroidal fibroblasts and retinal microvascular endothelial cells, accompanied with inhibited collagen gel contraction in vitro [105]. In neovascular AMD mice, local administration of recombinant IL-33 was also proved to suppress choroidal neovascularization formation via ST2 signaling. Notably, a recent study showed distinct findings. In the plasma of neovascular AMD patients, IL-33 level did not differ significantly compared to those in healthy controls [106]. Thus, more clinical and experimental research is required to elucidate this discrepancy. Also, comparing the expression and functional differences of IL-33/ST2 between different types or severity of AMD will not only help us better understand the complex mechanism of the disease but also provide new clues about the immunotherapeutic treatment paradigm.

In patients with PCV, plasma IL-33 level was as twice as high compared with that in the healthy people, together with diminished Tregs and enhanced polarization into Th2-like Tregs [106]. Meanwhile, the increased IL-33 was positively correlated with the high percentage of Th2-like Tregs in the peripheral blood. Interestingly, in the subgroup of PCV patients who could be classified as pachychoroid neovasculopathy, similar results were obtained. Taken together, IL-33 may participate in the immunologic dysfunction in PCV and pachychoroid neovasculopathy. However, there are numerous issues remain unknown, such as the mechanism and causality of IL-33/ST2 in PCV and the difference between IL-33/ST2 in the pachychoroid neovasculopathy and neovascular AMD.

One recent study also found that IL-33/ST2 might exert protective roles in RD [107]. In healthy mice, the expression level of IL-33 and several inflammatory mediators (IL-1*β*, IL-18, CCL2, complement components C1ra and C1s, and glial fibrillary acidic protein) quickly increased in the detached retina after the induction of RD and reduced gradually. However, in IL-33 knockout RD mice, an exacerbated and sustained inflammatory response together with persistent retinal gliosis were detected. These aggravated immune actions consequently led to a more severe photoreceptor degeneration and synaptophysin loss, when compared to control mice. Interestingly, IL-33 deletion suppressed the expression of CCL2 and IL-6 in Müller cells, whereas promoted the expression of proinflammatory IL-1*β*, CCL2, tumor necrosis factor-*α*, and nitric oxide synthase in macrophages.

In the context of ROP, serum level of IL-33 was increased in ROP infants who need laser therapies and was found to greatly reduced after the treatment. On the other hand, in the cord blood, IL-33 value was similar in ROP infants and healthy infants. Hence, serum IL-33 is preferred as a novel and sensitive biomarker for the prediction of severe ROP [108].

Nevertheless, IL-33 was rarely detectable in the serum, vitreous, and aqueous humor samples collected from patients with PDR. Moreover, the level of IL-33 did not alter significantly in all of these bodily fluids [109, 110].

There is also one research showed that IL-33 was significantly increased in the serum of NMOSD patients and was associated with disease status and relapse rate [111].

## 3. Discussion and Conclusions

We now have a deeper understanding about the expression, signaling pathway, and immunoregulatory mechanism of IL-33/ST2 axis in different parts of the eye. The current studies reaffirmed the previous findings that were obtained in other body systems or organs and extended to a broader field, involving a greater variety of cell types and immune responses. IL-33/ST2 may exert detrimental effects on allergic eye disease and corneal fungal infection, while play protective roles in bacterial keratitis, corneal wound healing, and RD. IL-33/ST2 also regulates the immune responses in DED, OT, AMD, PCV, ROP, and NMOSD. However, the deterioration/amelioration effects and specific signaling pathways of the axis still require further elucidation among these diseases. Additionally, whether IL-33/ST2 participates in uveitis and PDR remains unclear.

Apart from the unsolved questions stated in the abovementioned diseases, there is still much to be identified in future studies. For instance, genome variations are considered as important factors contributing to numerous autoimmune and inflammatory diseases. Nevertheless, few studies have reported the association between IL-33 and ST2 with ocular diseases in gene level to date. Besides, little is known about the role of IL-33/ST2 in corneal allograft rejection, considering it has been documented that IL-33/ST2 regulates the graft versus host disease-related immune responses in various organs. In summary, IL-33/ST2 is a promising axis in ocular diseases, which deserves more and consistent attention.

## Figures and Tables

**Table 1:** Involvement of Interleukin-33 (IL-33)/suppression of tumorigenicity 2 (ST2) axis in ocular diseases.

Disease	Findings	Refs
Allergic eye disease	IL-33 induced recruitment and ST2 expression of eosinophils and promoted T helper 2 (Th2) cytokines expression in allergic conjunctivitis (AC) mice model.	[73, 81]
	IL-33 was upregulated in the conjunctival giant papillae of atopic keratoconjunctivitis (AKC). IL-33/ST2 axis induced the phosphorylation of p38 mitogen-activated protein kinases (MAPK) and IL-13 mRNA induction in mast cells.	[74]
	The induction of proallergic IL-33 was triggered by specific Toll-like receptors (TLR) ligands through innate immunity signaling pathways in corneal epithelium.	[75]
	IL-33/ST2 mediated proallergic responses via nuclear factor kappa B (NF-*κ*B) signaling pathway and induced the production of proallergic cytokines and chemokines in human corneal epithelial cells (HCECs).	[76]
	Overexpression of IL-33 in keratinocytes induced the onset of AKC, accompanying the activation of corneal group 2 innate lymphoid cells (ILC2) and the release of Th2 cytokines.	[77]
	IL-33 mRNA and protein increased in conjunctival epithelial tissue in AC mice. Eosinophil and basophil infiltration and Th2 cytokines expression decreased in the IL-33 knockout mice.	[78, 79]
	IL-33 augmented CD4+, ST2+, or IL1RAP+ cells infiltration and Th2 cytokines expression in AC mice model. Clinical and molecular changes decreased in TLR4 deficient or MyD88 knockout mice.	[80]
	Total IgE concentration in serum and mast cells infiltration in conjunctiva increased in AC mice model.	[81]

Keratitis and corneal regeneration	ST2 mRNA and protein levels elevated in the *Pseudomonas aeruginosa*- (*P. aeruginosa*-) infected cornea in Th-2 responsive mice. Blocking ST2 signaling led to susceptible to *P. aeruginosa*-induced keratitis.	[83]
	IL-33 mRNA and protein levels elevated in the *P. aeruginosa*-infected cornea in Th-2 responsive mice. Stimulating IL-33 signaling led to resistance to *P. aeruginosa*-induced keratitis.	[84]
	IL-33 mRNA and protein levels elevated in the *Aspergillus fumigatus*-infected cornea in human, mice, and HCECs. IL-33/ST2/p38 MAPK axis amplified the proinflammatory responses induced by *Aspergillus fumigatus* in HCECs.	[85]
	IL-33/ST2 induced the production of inflammatory mediators (tumor necrosis factor-*α*, IL-1*β*, IL-6, and IL-8), while ST2 antibody, soluble ST2, NF-𝜅B activation inhibitor and inhibitor of NF-*κ*B inhibitor suppressed the signaling.	[86]
	IL-33 increased in cornea tissue after corneal epithelial wounding, promoting the number and function of corneal ILC2s in the healing process.	[87]

Dry eye disease (DED)	IL-33 and ST2 protein increased in the conjunctival impression cytology of DED patients. IL-33 mRNA and protein elevated in DED cell model.	[89]
	IL-33 concentration elevated in tears of DED patients.	[90]
	Elevated IL-33 level in tears of DED patients was positively related to increased Th2 cytokines (L-4, IL-5, and IL-13) and clinical severity.	[89, 91]

Uveitis	Upregulated IL-33/ST2 axis promoted macrophages polarization, altered the cytokines production profile, and reduced the disease severity in autoimmune uveitis mice.	[93]
	Serum IL-33 level increased in Behçet uveitis patients compared to Behçet patients without uveitis.	[94]
	A single nucleotide polymorphism-rs3773978 in gene interleukin 1 receptor like 1 significantly was associated with acute anterior uveitis (AAU) in Chinese population.	[95]
	No differences of IL-33 level in aqueous humor and serum were found among HLA-B27 associated AAU patients, idiopathic AAU patients, and control group.	[96]

Vitreoretinal diseases	Increased IL-33/ST2 signaling was closely correlated with the enhanced expression levels of IL-1*β*, Th1, and Th2 cytokines in eyes of *Toxoplasma gondii*-infected mice.	[97]
	Upregulated mRNA levels of triggering receptor expressed on myeloid cells-1 and TLRs were correlated with increased IL-33/ST2 signaling in damaged retina and choroid of *Toxoplasma gondii*-infected mice.	[98]
	IL-33 was highly released by Müller cells in the lesion area in age-related macular degeneration patients. Upregulated IL-33/ST2 signaling in Müller cells resulted in downstream cytokines and chemokines release, photoreceptor cell loss, and mononuclear phagocytes recruitment after light exposure.	[101]
	IL-33 and ST2 expression elevated in retinal pigment epithelium cells of age-related macular degeneration cell model. IL-33/ST2 signaling induced inflammatory cytokines secretion via MAPK, c-Jun N-terminal kinase, extracellular regulated protein kinases, NF-*κ*B pathways.	[102]
	IL-33 production, retinal vascular progression, and subretinal fibrosis formation decreased after antiendoglin and/or antivascular endothelial growth factor-A antibody therapy.	[103]
	IL-33/ST2 was upregulated by Toll-like receptor activation or hypoxic condition and can subsequently protect retina degeneration and choroidal neovascularization development.	[105]
	Plasma IL-33 increased in patients with polypoidal choroidal vasculopathy, together with diminished Tregs and enhanced Th2-like Tregs. The level of IL-33 was positively correlated with the percentage of Th2-like Tregs.	[106]
	IL-33 increased in the detached retina in mice. IL-33 deficiency resulted in exacerbated and sustained retinal inflammation, together with enhanced retinal degeneration and gliosis.	[107]
	Serum IL-33 increased in infants with retinopathy of prematurity, and greatly reduced after laser treatment.	[108]
	No significant changes were observed in both percent detectable and level of IL-33 in the serum, vitreous, and aqueous humor of proliferative diabetic retinopathy patients.	[109, 110]

Neuromyelitis optica spectrum disorder	Upregulated level of serum IL-33 was associated with disease status and relapse rate in neuromyelitis optica spectrum disorder.	[111]
